# Maternal Circulating Vitamin Status and Colostrum Vitamin Composition in Healthy Lactating Women—A Systematic Approach

**DOI:** 10.3390/nu10060687

**Published:** 2018-05-28

**Authors:** Jasmijn Y. de Vries, Shikha Pundir, Elizabeth Mckenzie, Jaap Keijer, Martin Kussmann

**Affiliations:** 1 Human and Animal Physiology, Wageningen University, De Elst 1, Building 122, 6708 WD Wageningen, The Netherlands; jasmijn.yamile@gmail.com; 2 Liggins Institute, University of Auckland, 85 Park Road, Grafton, Private Bag 92019, Auckland 1142, New Zealand; liz.mckenzie@auckland.ac.nz (E.M.); m.kussmann@auckland.ac.nz (M.K.); 3 Human and Animal Physiology, Wageningen University, De Elst 1, Building 122, 6708 WD Wageningen, The Netherlands; jaap.keijer@wur.nl; 4 New Zealand National Science Challenge “High-Value Nutrition”, University of Auckland, Auckland 1142, New Zealand

**Keywords:** human milk, vitamins, colostrum, plasma, infant

## Abstract

Colostrum is the first ingested sole nutritional source for the newborn infant. The vitamin profile of colostrum depends on the maternal vitamin status, which in turn is influenced by diet and lifestyle. Yet, the relationship between maternal vitamin status and colostrum vitamin composition has not been systematically reviewed. This review was conducted with the aim to generate a comprehensive overview on the relationship between maternal serum (plasma) vitamin concentration and corresponding colostrum composition. Three electronic databases, Embase (Ovid), Medline (Ovid), and Cochrane, were systematically searched based on predefined inclusion and exclusion criteria. Finally, a total of 11 eligible publications were included that examined the vitamins A, C, D, E, and K in both biological fluids. Maternal vitamin A, D, E, and K blood levels were unrelated to colostrum content of the respective vitamins, and serum vitamin A was inversely correlated with colostrum vitamin E. Colostrum versus maternal serum vitamins were higher for vitamins A, C, and K, lower for vitamin D, and divergent results were reported for vitamin E levels. Colostrum appears typically enriched in vitamin A, C, and K compared to maternal serum, possibly indicative of active mammary gland transport mechanisms. Inter-individual and inter-study high variability in colostrum’s vitamin content endorses its sensitivity to external factors.

## 1. Introduction

Exclusive breast feeding is recognised as the normative standard of infant feeding for the first six months of life, with continued breastfeeding for one to two years of life, or longer [1,2]. Human milk uniquely fits the human infant requirement, containing both nutritive and non-nutritive bioactive factors that promote survival and healthy development [3]. This biological fluid produced by the mammary glands has a rather complex composition, containing macronutrients (fats, carbohydrates, proteins), micronutrients (vitamins and minerals), protective factors e.g., lactoferrin, immunoglobulins and lysozymes, and development-relevant components, such as cytokines, growth factors, oligosaccharides, and hormones. Its composition is subject to dynamic changes within one feeding, with time of the day, over the lactation period, and between mothers and populations. In addition, it is influenced by maternal genetic and environmental factors, such as infection status [4] and maternal lifestyle, including dietary habits [5,6].

### 1.1. Colostrum, Transitional- and Mature Milk

Human milk composition changes as lactation progresses, and based on the composition, the lactation period is divided into three different stages: Colostrum, transitional-, and mature milk. Colostrum is the first fluid produced by the mother after delivery, and is distinct in appearance, composition, and volume. The first days postpartum, colostrum appears typically thick with a yellow hue and is produced in low quantities. Colostrum is rich in developmental factors, such as epidermal growth factor, as well as immunologic components, such as secretory IgA, lactoferrin, leukocytes [7,8,9]. In addition, it contains high amounts of protein, vitamin A, vitamin B12, and vitamin K [10], and low levels of lactose. As tight junction closure occurs in the mammary epithelium, a decline in sodium-potassium ratio and an incline in lactose concentration takes place, indicating secretory activation and transitional milk production. Transitional milk shares some of the characteristics of colostrum, but stands for a period of increased milk production that supports nutritional and developmental requirements of the rapidly growing infant from five days to two weeks postpartum. After the first two weeks, the milk is considered largely mature. By four to six weeks postpartum, the fully mature stage is reached, after which the human milk composition remains relatively constant with only subtle changes occurring over the course of lactation.

### 1.2. Nutritional Constituents of Human Milk

Human milk nutritional components come from three sources: Maternal diet, maternal stores, and lactocyte nutrient production. Fats, oligosaccharides, and proteins compose the major digestible energy components of human milk. The mean macronutrient composition of mature, term milk is estimated to be approximately 0.9 to 1.2 g/dL for protein, 3.2 to 3.6 g/dL for fat, and 6.6 to 7.8 g/dL for lactose [11,12,13,14]. In 1991, Nommsen et al. demonstrated that macronutrient concentrations of human milk at four months postpartum primarily depend on the maternal factors including body weight for height, protein intake, parity, return of menstruation, and nursing frequency [11]. Vitamins and essential minerals and trace elements, together termed as micronutrients, are present in human milk with varying concentrations throughout lactation, ideally suiting the infant.

### 1.3. Vitamins, Vital to Life

Vitamins are defined as organic dietary compounds required by the human body in relatively small, yet essential quantities, for healthy growth, development and to sustain life. They are a group of compounds with functions ranging from essential cofactors for numerous enzymes to key regulators of gene expression and to antioxidant function. They are important for metabolic processes, mineral homeostasis and bone development, vision, cognitive function, cardiovascular health, and immunity [15,16,17]. A lack of vitamins results in overt symptoms of deficiency related to these processes. Vitamins vary in polarity, which determines their way of transport through the body. The lipid-soluble vitamins A, D, E, and K require carrier proteins or lipoprotein vesicles for transport via the blood, whereas water soluble vitamins, such as thiamin (B1), riboflavin (B2), niacin (B3), pyridoxine (B6), cobalamin (B12), folate, biotin, panthothenic acid, and ascorbic acid (vitamin C), either circulate in blood freely or are bound to a carrier protein, with vitamin B6 and B12 as two examples.

### 1.4. Maternal Nutritional Status and Milk Vitamin Composition

The gestational period is nutritionally critical for maternal, foetal, and infant health. During these nine months, the mother provides the nutritional needs of the foetus and prepares for lactation by building up nutrient stores. In the subsequent exclusive breastfeeding period, lactating mothers are vulnerable to vitamin deficiency as the newborn feeds on her stores through milk ingestion. Accordingly, the dietary reference intakes (DRIs) of multiple vitamins are higher for lactating women, as set by the Food and Nutrition Board of the Institute of Medicine (Table 1). Reference intakes have also been established by several national agencies and by the Food and Agriculture Organization/World Health Organization/European Food Safety Authority (FAO/WHO/EFSA); these values tend to be lower than the recommended daily allowances (RDAs) [18]. Adequate intake (AI) of vitamins in the first six months of life is calculated based on average intake values via maternal milk (Table 1).

### 1.5. Factors Influencing Vitamin Status of Mother and Infant

As vitamins are derived from the diet, the maternal dietary pattern is decisive for her vitamin status. The bioavailability of ingested vitamins in the organism is affected by several aspects, including physiological factors, such as nutritional status, amount of fat in the diet, food matrix, and interactions with other nutrients [19,20,21,22]. Lifestyle habits, such as smoking, the use of drugs, alcohol, and oral contraceptives, and nutritional malnourishment (body mass index (BMI) < 18 or BMI > 25), are known to negatively impact maternal vitamin status [23,24,25,26,27,28]. For some vitamins, placental transfer is limited during gestation and thus the neonate’s vitamin stores can become depleted [29,30,31]. Newborns therefore depend on vitamin-rich early breast milk to replenish to replenish vitamin stores, and vitamin-sufficient later milk to maintain vitamin sufficiency.

The maternal vitamin status is of crucial importance to sufficiently provide her newborn infant with vitamins, and micronutrient deficiency remains a critical health concern among pregnant and lactating women. Especially in the immediate post-partum period, the newborn’s vitamin stores need replenishment to assure healthy growth and development. Considering that colostrum is the sole nutritive source for the infant in this period, it is key to understand the relation between the maternal vitamin status and the colostrum vitamin profile. This information would allow for nutritional optimisation in the maternal-infant dyad.

Therefore, we conducted a systematic review, with the procedure as described by the Preferred Reporting Items for Systematic Reviews and Meta-Analyses (PRISMA) statement of Systematic Reviews being followed up to the quality assessment stage, on the relationship between serum/plasma vitamin levels as a proxy for maternal vitamin status and colostrum vitamin composition in healthy lactating women of term infants, and to what extent the maternal vitamin status is translatable to human milk vitamin composition. We focused on healthy women and term infants in order to identify baseline data. No systematic review exists on the relationship between maternal serum (plasma) vitamin composition and human milk (colostrum) vitamin content. Available reviews have only investigated the impact of maternal status on serum levels of single vitamins. The amount of scientific articles assessing both maternal vitamin status and colostrum vitamin composition in healthy mothers of term infants is expected to be limited, since the vast majority of studies pertain to malnourished, vitamin-deficient populations, non-simultaneous blood and milk collection, and infant health outcomes. Our systematic approach therefore fills a gap in the current knowledge.

## 2. Literature Search Methods

The databases Embase (Ovid; 1980), Medline (Ovid; 1946), and Cochrane Central Register of Controlled Trials were searched up to 11 October 2017 to identify studies examining the micronutrient composition of maternal plasma and breast milk. The search was conducted by using combinations of the terms “human milk”, “breast milk”, “lactation”, “micronutrients”, “serum”, and the name of each vitamin and essential mineral. In addition, the search was limited to the English language and term delivery. Search terms were adjusted according to the different databases using appropriate terms and truncation marks. Studies in humans published as full-length articles were selected. To identify any other relevant publications, the electronic search was expanded by searching the reference lists of the selected publications.

### 2.1. Exclusion and Inclusion Criteria

The aim of this project was to identify baseline data on the natural maternal micronutrient status and respective composition of human breast milk. Therefore, publications were excluded if they focused on participants that were confounded by known adverse conditions. These comprised mothers at higher risk of nutritional or metabolic disorders or with major chronic diseases (e.g., diabetes, hypertension, or HIV/AIDS); mothers that were smoking during pregnancy and/or lactation; populations classified as having a low socio-economic status (defined by low education level, employment and income) and reported drug or oral contraceptive use by mothers; malnourished (pre-pregnancy BMI < 18.5 or BMI > 25 [35]) or micronutrient deficient populations (as indicated by authors); children with health problems (e.g., low birth weight or atopic dermatitis); and preterm deliveries (<37 completed weeks of gestation). Case-control studies were excluded from the review because they represent extreme cases, rather than the natural range of a population. Secondary articles, review articles, editorials, as well as conference proceedings without published full text articles, were excluded. When multiple publications considered the same study population and outcome, we included only the most complete publication. Insufficient information on the health status of the study population was regarded a limitation of the publication. Due to the confined time frame of the current project, the review output was further restricted to vitamin composition in colostrum (first five days of lactation), wherever blood (plasma or serum) samples were also analysed for vitamins. Publications that only reported levels of pro-vitamins without quantifying the active vitamin itself were excluded.

Articles were included if they provided quantitative information on the relationship between maternal serum/plasma vitamin status and the vitamin composition of colostrum. The relationship between maternal serum/plasma vitamin status and colostrum composition was estimated or quantified by indicators including correlation coefficients, means within subgroups, and estimates from regression models or *p* values from comparison tests. Furthermore, studies were included only when maternal plasma and colostrum samples were reportedly obtained at the same time point. Prospective and retrospective studies (randomised controlled trials, randomised controlled clinical trials, cohort studies, longitudinal studies) were included if they investigated healthy lactating mothers of singleton infants. An overview of the inclusion and exclusion criteria is provided in the Table 2.

### 2.2. Data Extraction

Using Covidence software, two investigators independently reviewed titles, abstracts, and full-text articles and assessed all potential studies that were identified as a result of the search strategy and were selected based on inclusion and exclusion criteria. Results were then compared, and disagreements were resolved through discussion and consensus, or by consultation with a third investigator. The systematic review strategy was executed up to the point of quality assessment. Thereafter, the resulting articles were assessed and summarised in detail for this review. Information on study design, geographical area, description of participants (sample size, age of mothers, maternal and infant characteristics), blood and colostrum sample collection (number of samples, characteristics of collection and storage), vitamin composition of blood and colostrum, and quantitative estimates on the relation between maternal vitamin status and colostrum was extracted from each publication. Reported results on mixed populations regarding general, maternal, and newborn characteristics that did not comply with our inclusion criteria were denoted as limitations of the respective study (Table 3).

## 3. Results

**Figure 1:**
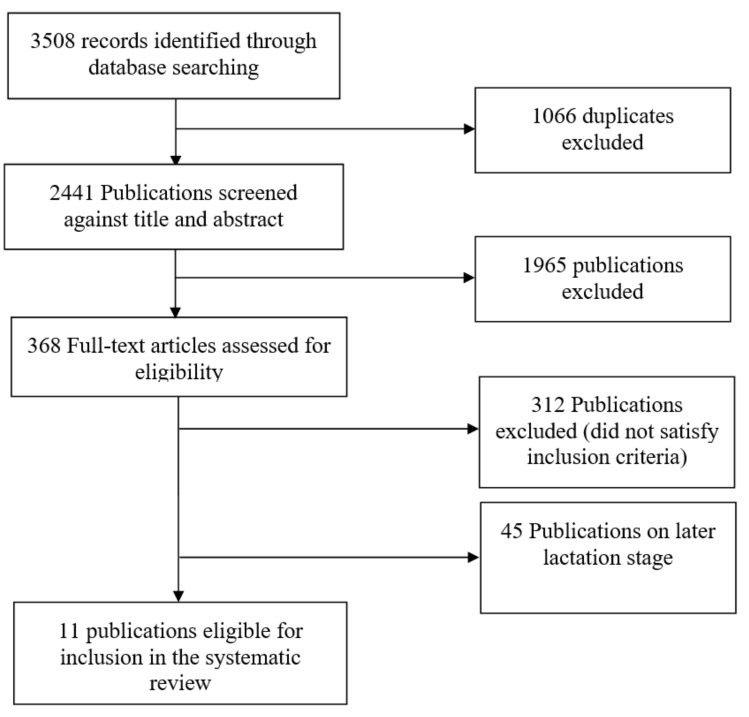
Publication selection procedure. The flow chart depicts systematic steps of title, abstract and full-text screening as independently conducted by two authors.

The publication selection procedure is depicted in Figure 1. The initial search yielded 3508 records, of which 1066 were duplicates. The remaining 2441 articles were evaluated based on their title and abstracts, excluding another 1965, because of being out of scope. The remaining 368 full texts were assessed for eligibility. Of these, 312 papers were removed that did not satisfy inclusion criteria, and only 11 articles remained eligible. Full texts of three articles that were found eligible based on their title and abstract were not available through the University of Auckland or Wageningen University libraries, and were therefore not incorporated in this review. Of all texts, one was excluded based on non-English language. No additional articles were obtained from the reference lists of the included articles. Hence, 11 articles were considered in the present review, of which six were cross-sectional studies [41,42,43,44,45,47], four described randomised controlled clinical trials [36,38,39,40], and one was a quasi-experimental intervention study conducted in a convenience sample without control group [48]. Coinciding collection of colostrum and blood were baseline measurements for all randomised controlled trials and the quasi-experimental study, thus data on lost to follow up were not extracted for the current review. One study was published in 1986, and the other 10 were published after 2001. Eight articles concerned studies conducted in South America (Brazil), two in Europe (France and The Netherlands), and one in South Asia (Bangladesh). The publications from Brazil originated from different departments of the Federal University of Rio Grande do Norte, Natal, with R. Dimenstein as corresponding author. One of them was conducted in cooperation with the Federal University Campina Grande (UFCG) [48], and one with Potiguar University (UnP), Laurate International Universities, Natal [38]. In total, 741 mothers were considered, and all studies included women of reproductive age, between 14–41 years.

### 3.1. Colostrum and Blood Vitamin Composition

In eight studies, colostrum was obtained by manual expression [36,39,41,42,43,44,47,48]; in two studies, a manual breast pump was used [45,40]; and in one publication, no information on expression method was given [38]. Blood sample collection was done by venipuncture in five studies [36,39,43,44,48]; one article stated blood samples concerned venous blood [40], and in five publications no information on blood collection method was given [38,41,42,45,47]. In the majority of the studies, colostrum and blood samples were obtained at one time point between the first and fifth postpartum day, except for one study collecting colostrum samples over 1–3 days postpartum in order to establish a colostrum pool [42], and for three publications, the postpartum day was not mentioned [36,43,48]. Nine articles indicated that the samples were stored at a temperature ranging between −18 °C and −70 °C after serum preparation [38,39,40,41,42,43,44,45,48]. In eight studies, samples were collected after an overnight fast [36,38,39,41,42,43,44,48]; whereas in the remaining three studies, the prandial state of participants was not specified [40,45,47]. Vitamin content of maternal serum/plasma and colostrum was analysed through high-performance liquid chromatography-UV (HPLC-UV) in eight studies [36,38,39,40,41,42,43,44], one combined HPLC-UV and a protein-binding assay [45], and one study used UV spectrophotometry [47]. With regards to the vitamins assessed, six publications examined vitamin A [38,41,42,43,44,48], five examined vitamin E [36,38,39,41,42], and vitamin C [47], D [45], and K [40] were examined by single articles. The majority of articles reported concentrations of a single vitamin [36,39,43,44,45,47,48], two publications reported both vitamin A and E levels [41,42], and one article described both vitamin K and E content in colostrum and plasma [40]. Vitamin D metabolites were quantified through isotopically labelled internal standards [45], and vitamin D was quantified by a competitive protein-binding assay, with the standard curve ranging from 0–13 to 0–26 pmol/assay tube [45]. Six studies used external standards to quantify vitamin A and E [36,38,41,42,43,48], and two studies used internal standards for vitamin A [44] and vitamins K and E [40]. In the publication by Ahmed et al., the degree of certainty of vitamin C quantification was not documented [47]. The correlation between maternal serum and colostrum vitamin levels as tested through linear regression analysis was reported by seven articles [39,40,41,42,44,45,48], and the remaining articles did not report this relationship through a statistical method [36,38,43,47].

Hereafter, results concerning the relationship between maternal blood/serum levels and colostrum vitamin concentrations are described, ordered by vitamin. All reported concentrations are given in mean ± SD, and reflect one time-point of both blood and colostrum collection, unless stated otherwise.

### 3.2. Vitamin C

Ahmed et al. [45] reported an eight-fold lower vitamin C concentration in maternal serum compared to the corresponding colostrum samples (0.44 ± 0.29 mg/dL versus 3.50 ± 0.49 mg/dL) of a subsample of seven participants. In the rest of the population (*n* = 19), no serum vitamin C measurement was performed. Serum vitamin C levels were within the normal range of 0.4–1.5 mg/dL [49]. The mean reported colostrum vitamin C concentration was in agreement with the reference value range of 3 to 10 mg/dL [47]. Clinical vitamin C deficiency symptoms occur when plasma levels are <0.2 mg/dL [50,51].

### 3.3. Vitamin D

Cancela et al. [44] reported mean concentration levels of Vitamin D 25-hydroxyvitamin D (25OHD), the major metabolically active form of vitamin D present in serum [52], and 1,25-(OH)_2_D_3_ to be lower in colostrum as compared to maternal serum. The (mean ± SEM) vitamin D and 25OHD concentrations in colostrum were 0.89 ± 0.32 nmol/L and 0.50 ± 0.11 nmol/L, respectively, and serum 25OHD and 1,25-(OH)_2_D_3_ were reportedly 22000 ± 2610 nmol/L and 0.194 ± 0.047 nmol/L, respectively, for a sample of 11 mothers. Maternal serum and colostrum vitamin D levels were individually analysed and reported for one mother that received vitamin D supplementation after postnatal day seven. Her mean serum 25OHD and 1,25-(OH)_2_D_3_ concentrations were 20,000 nmol/L and 0.204 nmol/L, respectively, and colostrum 25OHD and vitamin D were 0.44 nmol/L and 0.21 nmol/L, respectively. Maternal serum levels of neither 25OHD nor 1,25-(OH)_2_D_3_ correlated with the vitamin D or 25OHD content of milk of the same mother at 3–5 d postpartum [45]. Sufficient vitamin D levels in serum are set to a minimum of 20 ng/mL (50 nmol/L) 25OHD [53,54,55,56].

### 3.4. Vitamin K

Thijssen et al. [40] reported higher baseline phylloquinone concentrations in colostrum (5.84 ± 2.31 nmol/L) as compared to plasma levels (2.62 ± 1.91 nmoL/L), indicating a colostrum:plasma ratio of 3.97 ± 3.11. Baseline menaquinone-4 colostrum levels were higher (3.18 ± 1.53 nmoL/L) than in plasma (0.25 ± 0.16 nmoL/L), with a colostrum:plasma ratio reported of 14.83 ± 8.3. Menaquinone-4 was only detected in 10 of the total 31 plasma samples of participants. No correlation was found between plasma and milk levels of vitamin K. The reported colostrum and plasma distribution values, given as colostrum:plasma ratio, did not correspond to the indicated mean concentrations in both biological fluids in the article. The same study included vitamin E measurements for comparison reasons, but did not examine the interaction between plasma vitamin K and colostrum vitamin E, or vice versa. The major circulating form of vitamin K is phylloquinone, and serum values in healthy adults ranging from 0.2 to 1.0 μg/L, with a median of around 0.5 μg/L, are considered sufficient [57].

### 3.5. Vitamin E

Thijssen et al. [40] reported colostrum α- and γ-tocopherol concentrations to be slightly lower than in plasma (1141.3 ± 796.7 µg/dL compared to 1309.2 ± 262.7 µg/dL), indicating a colostrum:plasma ratio of 0.97 ± 0.62. γ-Tocopherol is a naturally occurring form of vitamin E. It is a potent antioxidant [58], but the human body preferentially retains α-tocopherol in the liver, distributes it to extrahepatic tissues, and actively metabolises and excretes other forms of vitamin E. 

Similarly, Garcia et al. [41] established α-tocopherol concentrations to be slightly lower in colostrum (1217.4 ± 959.9 µg/dL) in comparison to plasma (1326.9 ± 278.2 µg/dL) in 73 vitamin E adequate mothers considering serum α-tocopherol reference values (>697.7 µg/dL [59]). No reference values for colostrum α-tocopherol adequacy were mentioned.

De Lira et al. [42] reported comparable α-tocopherol levels in colostrum (1124.0 ± 551.2 µg/dL) and serum (1137.0 ± 344.5 µg/dL), in a population of 103 mothers with a subclinical vitamin E deficiency prevalence of 16% after individual analysis. In the vitamin E adequate (≥697.7 µg/dL [37]) subgroup, α-tocopherol concentrations were similar in colostrum (1216.2 ± 693.4 µg/dL) and serum (1236.0 ± 348.0 µg/dL). Of this subgroup, subclinical α-tocopherol deficiency in colostrum, defined as levels <1124.0 µg/dL, was detected in 44% of the cases. As no reference value for colostrum α-tocopherol is available from literature, the authors used the mean colostrum α-tocopherol concentration of those individuals who exhibited adequate levels of serum α-tocopherol in the respective study, as cut-off for adequacy.

Melo et al. [36] reported baseline α-tocopherol concentrations in colostrum for two similar groups to be higher in colostrum (1509.3 ± 793.7 mg/dL and 1452.9 ± 808.6 mg/dL) than in serum (1066.6 ± 287.7 mg/dL and 1159.6 ± 350 mg/dL). Of the entire population (*n* = 99), 6.1% had serum levels indicating low levels of α-tocopherol (ranging from 499.6 to 697.7 mg/dL), while 3.0% had α-tocopherol deficiency (levels < 499.6 mg/dL) [37]. The concentrations appear to have been erroneously reported as mg/dL, but are likely to be in µg/dL, in line with other published studies and calculations regarding nutritional needs in this publication. Contacting correspondent author R. Dimenstein did not clarify this issue. The concentrations were incorporated as µg/dL in the current review.

Clemente et al. [39] reported higher baseline colostrum α-tocopherol for three homogenous groups (1665.2 ± 160.2 µg/dL, 1387.1 ± 176.5 µg/dL, and 1802 ± 208.1 µg/dL), than respective concentrations in serum (1016 ± 52 µg/dL, 1236 ± 51 µg/dL, and 1083 ± 61 µg/dL). Low serum α-tocopherol levels (between 499.6 and 697.7 µg/dL) were reported for 6.6% of the total population (*n* = 109), but none of the participants had α-tocopherol deficiency (α-tocopherol < 499.6 µg/dL [37]).

Grilo et al. (2016) [38] found mean baseline α-tocopherol concentrations in colostrum for two homogenous groups to be higher (1189.4 ± 660.6 µg/dL and 1259.2 ± 608.2 µg/dL) compared to serum (1023.6 ± 380.4 µg/dL and 1059.0 ± 400.3 µg/dL), respectively. Individual serum α-tocopherol analyses showed that 9.1% of the participants had vitamin E deficiency (<516 µg/dL [19]). At baseline, the studied population (*n* = 88) was considered homogenous based on maternal, obstetric and newborn characteristics, and serum α-tocopherol and retinol levels.

**Figure 2:**
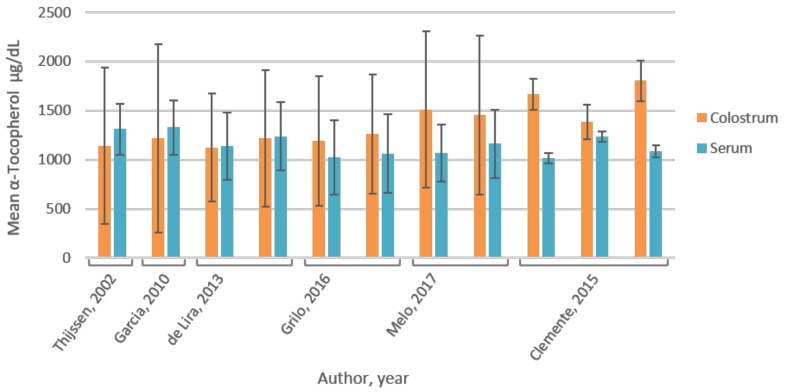
Reported α-tocopherol concentrations in colostrum and serum. Standard deviations for mean α-tocopherol levels in colostrum are major compared to serum in all publications. Mean concentrations of colostrum α-tocopherol are slightly lower than in serum reported by Thijssen and Garcia et al. [41,40]. No statistical significance of this difference was indicated and standard deviations show overlap. Reported α-tocopherol levels by de Lira et al. [42] are similar in the two biological fluids. Grilo and Melo et al. [36,38] reported higher mean colostrum α-tocopherol in colostrum compared to serum with overlapping standard deviations. Mean concentrations of α-tocopherol are significantly higher in colostrum compared to serum for two of the three groups studied by Clemente et al. [39].

No correlation between plasma/serum and colostrum vitamin E concentrations exists, according to Thijssen [40], Garcia (*r* = 0.74 and *p* = 0.54) [41], de Lira (*r* = −0.12, *p* = 0.22) [42], and Clemente et al. (*r* = 0.073, *p* = 0.440) [39]. Figure 2 provides an overview of the reported vitamin E values in serum and colostrum. Mean α-tocopherol concentrations in colostrum show large standard deviations as compared to serum within each of the studies. Differences in mean vitamin E concentrations between colostrum and serum showed no overlap in standard deviations for two of three groups of the study by Clemente et al. [39].

The eligible publications described vitamin E deficiency according to different reference values based on different sources. To test vitamin E adequacy, haemolysis has been used in subjects thought to be at risk for vitamin E deficiency, using hydrogen peroxide or other oxidants added in vitro. The biomarker for vitamin E deficiency selected by the Institute of Medicine in 2000 [19] was the plasma α-tocopherol concentration > 516 µg/dL, associated with normal in vitro hydrogen peroxide-induced hemolysis to 12 percent or less, as reported by Horwitt et al. (1963) [60]. In the latter study, the average α-tocopherol serum concentration in six subjects with haemolysis values of 12 percent or less was 697 µg/dL. In a review on laboratory tests for the assessment of nutritional status, Sauberlich et al. (1973) stated that when the serum α-tocopherol concentration was above 500 µg/dL, “*appreciable haemolysis was rarely observed*” [37]. A cut-off value for colostrum vitamin E deficiency was arbitrarily set by one eligible publication based on internal data [42].

### 3.6. Vitamin A

Grilo et al. (2016) [38] found higher baseline retinol concentrations in colostrum for two homogenous groups (101.3 ± 63.3 µg/dL and 102.1 ± 47.7 µg/dL) as compared to serum (44.8 ± 16.4 µg/dL and 48.3 ±15.4 µg/dL, respectively). Individual serum retinol analysis indicated 5.7% of the women in the entire study sample (*n* = 88) had vitamin A deficiency with levels <0.20 µg/dL [61]. No cut-off values for colostrum retinol deficiency were specified. At baseline, the studied population was homogenous based on maternal, obstetric and newborn characteristics, and serum retinol and α-tocopherol levels.

Garcia et al. [41] reported higher retinol concentrations in colostrum for two groups (74.49 ± 54.44 µg/dL and 68.77 ± 68.77 µg/dL), compared to retinol plasma levels (50.72 ± 14.33 µg/dL) in the total population of 73 participants. Plasma retinol levels were found adequate (≥0.30 µg/dL [62]). Colostrum retinol concentrations below 60 µg/dL [63] were considered indicative of a low retinol concentration, however the prevalence of deficiency was not indicated.

De Lira et al. [42] reported retinol in colostrum to be enriched compared to serum (62.46 ± 22.92 µg/dL versus 42.69 ± 11.46 µg/dL) in 103 mothers. By using a serum cut off value of ≥30 µg/dL [61] for retinol adequacy, individual analysis showed a moderate retinol deficiency rate of 15.5%, whereas 50% exhibited insufficient colostrum levels (<60 µg/dL [63]). A vitamin A adequate subgroup (*n* = 87) with serum retinol levels ≥ 30 µg/dL also exhibited higher colostrum concentration (64.47 ± 22.64 µg/dL) than serum (46.13 ± 10.03 µg/dL). In the subgroup, colostrum retinol levels suggestive of subclinical deficiency were detected in 34%.

Gurgel et al. [43] investigated the effect of prenatal multivitamin supplementation with different vitamin A sources (retinol + β-carotene, β-carotene-only, or retinol-only), including a control group receiving no supplementation (*n* = 25 for each group). Retinol concentrations below 20 µg/dL [64] in serum and below 60 µg/dL [61] in colostrum were considered indicative of vitamin A deficiency. All groups met the recommended dietary allowance of vitamin A intake for pregnant women (770 µg/d [32]), as established through food frequency questionnaires. In the control group, colostrum retinol concentrations were higher (96.6 ± 53.5 µg/dL) than serum (45.4 ± 11.8 µg/dL), with serum deficiency in 12% and colostrum inadequacy in 20%. For the group supplemented with retinol + β-carotene, colostrum retinol was reportedly higher than serum (126.1 ± 48 µg/dL versus 46.5 ± 13.3 µg/dL, respectively), with colostrum retinol inadequacy detected in 4%. The β-carotene-only supplemented group had higher colostrum retinol concentrations (89 ± 61.9 µg/dL) compared to serum (43.5 ± 13.7 µg/dL), with colostrum retinol inadequacy in 40%. The group that received multivitamin supplementation with only retinol as a vitamin A source exhibited higher colostrum retinol concentrations (136.8 ± 51.7 µg/dL) than serum (47.5 ± 13.0 µg/dL), with one case (4%) of colostrum retinol below adequacy level. In the supplemented groups, no maternal vitamin A inadequacy was established.

In a sample of 86 mothers, Da Silva et al. [44] reported colostrum retinol concentrations to be higher (100.3 ± 54.4 µg/dL) than in serum (28 ± 14.6 µg/dL), with retinol levels indicative of vitamin A deficiency (<20 µg/dL [62]) in 9.3% and colostrum retinol inadequacy (≤60 µg/dL [63]) in 22.1%.

Grilo et al. (2015) [48] reported median (range) retinol concentrations to be higher in colostrum 46.8 µg/dL (29.7 to 158.9), compared to serum 37.3 µg/dL (16.8 to 62.2). Median retinol concentrations in serum were considered adequate (≥20 µg/dL [62]), and no adequacy cut-off value for colostrum retinol levels was indicated.

**Figure 3:**
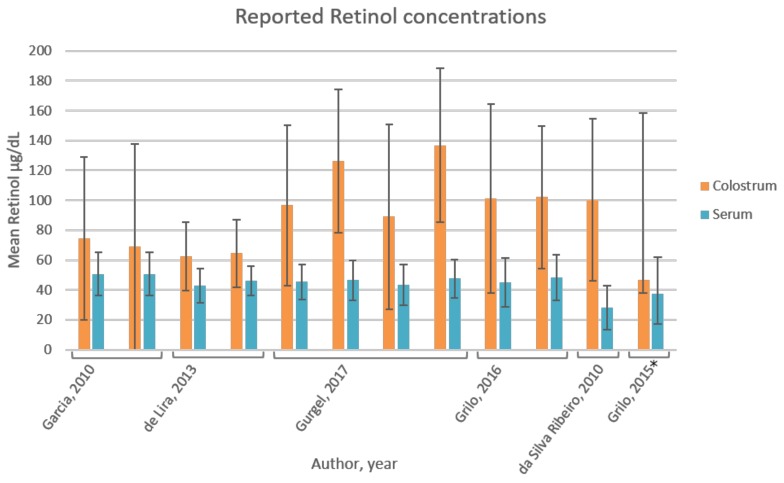
Reported retinol concentrations in colostrum and serum. All publications reported mean concentrations of retinol in colostrum to be higher than in serum. The colostrum retinol content was significantly higher in the study by da Silva Ribeiro et al. [44], for one of the groups by Grilo et al. (2016) [38], and for two groups by Gurgel et al. [43], compared to serum levels. The standard deviations of retinol levels in colostrum are large compared to serum. * Bars and error bars represent median and range retinol values, respectively.

There was no correlation between colostrum and plasma retinol according to Garcia (*r* = 0.39 and *p* = 0.74) [41], de Lira (*p* = 0.11, *r* = 0.15) [42], da Silva [44], and Grilo et al. (2015) [48]. An overview of the reported mean retinol values in serum and colostrum is depicted in Figure 3. All publications reported mean concentrations of retinol in colostrum to be higher as compared to serum. Mean retinol concentrations in colostrum show higher standard deviations as compared to serum within each of the studies.

The eligible publications included in the present review used different cut-off values to describe vitamin A status, as indicated per article. The cut-off value of 30 µg/dL serum retinol has been retained to indicate a low vitamin A status. In 2009, the WHO defined serum (plasma) retinol levels < 20 µg/dL indicative of vitamin A deficiency in pre-school children and pregnant women [64]. Among women, two retinol concentration cut-offs were used to estimate vitamin A deficiency and low-to-deficient vitamin A status, respectively: <20 µg/dL and <30 µg/dL [61,62,65]. Vitamin A deficiency was defined as occurring where tissues are depleted to a level of functional significance, even if this is not clinically evident. Presumably, this occurs when blood levels are below homeostatic set-points that respond to improvement in vitamin A status. There is no direct evidence of the serum cut-off value where functional consequences, morbidity/mortality effects, begin to occur.

As for colostrum vitamin A deficiency, four of the eligible publications [41,42,43,44] consistently considered colostrum retinol values lower than 60 µg/dL indicative of a low retinol concentration, referring Macias et al. 2001 [63] or West et al. 2002 [61], without defining or reporting criteria for this assessment. The referred articles do not provide this value. The WHO (1996) selected a cut-off value of ≤30 µg/dL breast milk based on dietary requirements of the infant to provide enough to prevent subclinical deficiency in the first six months of life [62].

### 3.7. Effect of (Serum) Retinol on Colostrum α-Tocopherol

Biochemical analysis by de Lira et al. of retinol-sufficient (≥30 µg/dL [61]) lactating women showed serum retinol concentrations to inversely correlate with α-tocopherol colostrum levels (*r* = −0.28, *p* = 0.008), but this was not observed for inadequate mothers with retinol serum levels below 30 µg/dL [42]. In line with this observation, Grilo et al. (2016) reported colostrum α-tocopherol levels to significantly decrease by 16.4% 24 h after supplementation with 20,000 IU liquid retinyl palmitate, presenting a negative impact of vitamin A supplementation on α-tocopherol colostrum levels [38]. In contrast, Garcia et al. pointed out a mega-dose supplementation of 200,000 IU retinyl palmitate, plus 49.4 mg of all-rac-α-tocopherol, to not negatively affect colostrum α-tocopherol levels after 24 h in vitamin A sufficient individuals (≥30 µg/dL), as the supplementation resulted in a significant increase of colostrum α-tocopherol [41]. This possibly indicates the all-rac-α-tocopherol contained in the supplementation capsule to counterbalance the negative impact of vitamin A supplementation on colostrum α-tocopherol content. As concluded by Garcia et al., the mega-dose of vitamin A did not negatively impact colostrum α-tocopherol [41]. In the latter two studies by Grilo and Garcia et al., no post-supplemental blood samples were obtained to consolidate a likely increase in systemic retinol levels, hence they did not directly assess an association between the reported colostrum α-tocopherol and serum retinol at this stage. The rest of the eligible publications did not report on interactions between vitamins.

## 4. Discussion

The systematic search resulted in 11 eligible publications describing the relation between maternal vitamin D, E, A, K, and C status and respective colostrum vitamin concentrations in healthy lactating mothers, with most papers focussing on vitamins A and E. As for vitamin E, varying results were reported on differences between mean colostrum and serum (plasma) levels [36,38,39,40,41,42]. Colostrum was consistently reported to be enriched in vitamin A compared to serum in six studies [38,41,42,43,44,48]. Regardless of the differences between mean concentrations of the two biological fluids, all publications established much higher standard deviations for colostrum vitamins as compared to serum content. Garcia et al. [41] claimed a positive overtime effect of mega-dose vitamin A supplementation on α-tocopherol colostrum content, whereas an inverse correlation between serum retinol and colostrum α-tocopherol levels was observed by de Lira et al. [42], which was further supported by the negative overtime effect of retinol supplementation on α-tocopherol colostrum reported by Grilo et al. (2016) [38].

We selected studies from well-nourished healthy lactating mothers from average-to-high socio-economic statuses, because the impact of maternal malnutrition was beyond the scope of our investigation. Furthermore, we excluded studies on preterm births, or pathological conditions, including low birth weight, atopic dermatitis, or infants born to HIV-infected mothers, because our aim was to investigate breast-milk composition under “healthy conditions”. This is, in fact, also a publication bias, regarding geographical area. Out of the total amount of eligible papers, eight were published by the same research group of Roberto Dimenstein and represented research conducted at different departments of the Federal University of Rio Grande do Norte, Natal, Brazil. The included articles from this group addressed vitamin A and/or E. This resulted in over representation of article addressing lipid-soluble vitamins A, D, E, and K, and water-soluble vitamin C. It may also be because newborn infants are particularly susceptible to vitamin A, D, E, and K deficiency as placental transfer to the foetus is limited, hence maternal milk vitamin content is crucial for the exclusively breast-fed infant. Primary reasons to address these vitamins were lacking information on blood-milk transfer mechanisms and reported controversy on adequate levels of these vitamins in human milk in the current literature.

Accurate measurement of vitamins in human milk is analytically challenging. Representative human milk samples must be collected from the population according to carefully defined procedures. Notably, the identified articles used various methods of human colostrum collection. Such differences in milk collection methods possibly underlie variation in reported vitamin content in colostrum between publications. Moreover, sample processing after collection and analytical methods of vitamin detection varied across included studies. Methodological factors that may affect colostrum vitamin level results are: Discarding the first milk ejection to avoid fluctuation in fat/vitamin content; use of one or two breasts; collection at the start or end of breastfeeding; prandial state of the mother; postpartum day; and to what degree of certainty the vitamins were quantified.

Incorporated articles on the relationship between maternal vitamin status and colostrum vitamin content exhibited limitations regarding the studied populations. There was heterogeneity in health, nutritional, obstetric, and general characteristics of the studied participants, both within and between publications. Papers varied in defining the maternal vitamin status, with authors using different reference values for both serum and colostrum vitamin adequacy. In addition, several articles lacked information on population characteristics (e.g., (pre-gestational) maternal BMI, newborn weight, socio-economic status, and absence-of-disease and/or health statement). For these reasons, reported data did not exclusively reflect the predefined inclusion and exclusion criteria for a vitamin-sufficient, healthy population, as we aimed to reveal. Nevertheless, cumulatively the reported data are of service to design research questions regarding maternal vitamin status and colostrum vitamin content, and in particular about the mechanism behind vitamin transport by the human mammary gland and strategies for nutritional modulation.

### 4.1. Correlation of Individual Vitamins in Blood and Colostrum

The relationship between serum and colostrum vitamin concentrations was analysed through linear regression analysis. Maternal serum levels of neither 25OHD nor 1,25-(OH)_2_D_3_ correlated with the vitamin D or 25OHD content of milk of the same mother [45]. Moreover, serum and colostrum levels of the vitamins K [40], E [39,40,41,42] and A [41,42,44,48] were not related. The absence of correlation between these fluids suggests the concentrations of individual vitamins in colostrum to be independent of respective serum content, which is most likely due to vitamin transport mechanisms to the mammary. Even though vitamin E [36,38], vitamin A [43,38], and vitamin C [47] concentrations in the referred publications were measured in both serum and colostrum, no statistical analysis on their correlation had been performed. Linear regression analysis assumes a linear response whereas in reality one component might influence another in a different fashion, in which case alternative statistical models should be used.

### 4.2. Vitamin C

An eight-fold enriched vitamin C content of colostrum compared to human serum was reported by Ahmed et al. [47]. The elevated concentration in breastmilk may be due to unique secretory activity in the mammary glands, suggesting “nature’s choice” to meet the newborn’s requirements. Vitamin C is an essential cofactor for a variety of metabolic reactions, has an important role in maintenance of redox homeostasis, and is necessary for the uptake and metabolism of several divalent minerals, especially iron. It has an important role in epigenetic regulation in the newborn infant, which may add to the explanation for enrichment of vitamin C in colostrum [66,67,68]. The active transport of vitamin C by both the placenta and human breasts is corroborated by the observation of foetal and infant vitamin C serum levels being 50% higher than those of the respective mothers [69,70]. In general, vitamin C concentration in milk varies greatly with values between 3 to 10 mg/dL, and depends on maternal dietary intake of the nutrient as well as other factors [70,71,72,73]. Ahmed et al. did not specify method of comparison, and, therefore, the degree of certainty to what the reported vitamin C concentrations were quantified is unknown.

### 4.3. Vitamin D

Mean concentrations of vitamin D and metabolites were lower in colostrum as compared to maternal serum as reported by Cancela et al. (1986) [45]. In addition, plasma and colostrum vitamin D and metabolite levels were reportedly unrelated. No other publications were identified directly evaluating this relationship at this stage of lactation. Later human milk displays lower vitamin D and 25OHD than maternal serum as well, but why vitamin D and its metabolites are transferred over the lactating mammary epithelium to the breast milk in such small quantities is unknown [74], and hence requires further investigation. Currently, serum 25OHD levels are regarded as the best biomarker of exposure, reflecting the net incoming contributions of cutaneous vitamin D synthesis and total intake [33,75], and the reported values by Cancela et al. were within the normal range in non-pregnant, non-lactating mothers [45].

Vitamin D and 25OHD are quantitatively the most important anti-rachitic sterols in human milk [76,77], and can be enriched through cutaneous vitamin D synthesis or high ingestion. Several studies in the 1980s demonstrated dietary vitamin D and ultraviolet or solar exposure to increase the vitamin D content in later human milk [78,79]. In addition, in 2004, Hollis and colleagues found high dose supplementation of vitamin D (2000 IU and 4000 IU) to significantly elevate the concentrations of both vitamin D and 25OHD in mature milk over a three month-intervention period, without reaching toxicity levels in maternal serum [80]. This possibly points to selective mammary gland uptake above a certain threshold.

Upon intestinal absorption, vitamin D is incorporated in chylomicrons and appears almost exclusively in the chylomicron fraction of plasma, whereas its metabolite 25OHD circulates the blood bound to vitamin D binding protein (DBP). Rapid postprandial disappearance of vitamin D from plasma is primarily due to uptake into adipose tissue and skeletal muscle, which mainly occurs through lipolysis [33]. Uptake pathways of vitamin D and 25OHD by the human mammary gland are yet to be specified, but could possibly be mediated by lipolysis and a specific 25OHD-DBP complex receptor [81], respectively.

### 4.4. Vitamin K

One eligible publication by Thijssen et al. reported vitamin K1 (phylloquinone) and K2 (menaquinone-4) levels in both colostrum and plasma, indicating colostrum levels of the vitamers to be higher than plasma levels [40]. Vitamin K is distributed through the body packed in triglyceride-rich very-low density lipoprotein (VLDL) fractions and chylomicrons [82,83]. The liver is the main storage site of phylloquinone, and hepatic phylloquinone uptake by endocytosis of chylomicron remnants is supposedly mediated by apolipoprotein E [82]. Whether and how extrahepatic distribution of these vitamers to peripheral tissues occurs is unknown [32]. Thijssen et al. found breast milk to be highly responsive to oral phylloquinone supplementation, given vitamin K content was further raised after oral phylloquinone supplementation measured in later milk [40]. This was also found by others [84,85,86] and suggests uptake by the mammary tissue may occur directly from chylomicrons as a result of intestinal phylloquinone absorption. Maternal phylloquinone supplementation could therefore be an alternative, more natural way to remedy the prevalent vitamin K deficiency in newborn infants, as compared to the intramuscular/subcutaneous phylloquinone injection that is generally administered to the infant [30,84,87].

The reported colostrum and plasma distribution values (colostrum:plasma ratio) by Thijssen et al. seem to be erroneously calculated, as they did not correspond to the indicated mean concentrations in both biological fluids in the article [40]. Plasma and colostrum vitamin K levels were reportedly unrelated, and no other publications were identified directly assessing this relation at this stage of lactation.

### 4.5. Vitamin A

Human milk vitamin A is derived from both maternal vitamin A stores (circulating plasma retinol bound to plasma retinol binding protein (pRBP) and transthyretin), as well as immediate dietary intake (transferred directly to the mammary gland from chylomicrons) [88,89,90]. However, the mechanism of vitamin A transfer to milk is yet to be fully understood in humans, and is being studied in animal models [91].

The reported high standard deviations (Figure 3) indicate high inter-individual variability of colostrum vitamin A content among the studied populations [38,41,42,43,44,48]. The absence of correlation between colostrum and serum retinol [42,44,48] signifies colostrum’s independence of maternal vitamin A status. This is likely because of the alternative chylomicron-mediated transport mechanism of retinol to the mammary gland during colostrogenesis [92,93]. Elevated lipoprotein lipase (LPL) activity in lactating mammary tissue may be responsible for hydrolysis of chylomicrons and uptake of retinol by the gland [94]. As further speculated by Green et al. [94], a large proportion of dietary vitamin A could be directed to the gland rather than to the liver, as opposed to the non-lactating state. Thus, active uptake of vitamin A from chylomicrons into the mammary tissues could explain the variability in colostrum retinol levels even when plasma retinol remains unchanged, characterising colostrum as particularly sensitive to maternal dietary intake of vitamin A. This proposed mechanism can possibly be extended to β-carotene, which is also taken-up in chylomicrons from the intestinal lumen. β-carotene is the main pro-vitamin A and accounts for a large proportion of dietary vitamin A intake, especially in vegetarians [95]. The β-carotene concentration in colostrum appears to be naturally high compared to later milk in healthy Swedish mothers [96], and is particularly sensitive to supplementation with purified β-carotene [97]. In transitional milk, supplementation with β-carotene did not lead to an increase in β-carotene concentrations [98], which might indicate mammary gland uptake is specifically enhanced at the immediate postpartum period. Purified β-carotene in oil has a six times higher RAE than in the dietary plant matrix [32]. β-carotene in lipid-rich colostrum therefore possibly serves as a major contributor to the newborn’s vitamin A status. No eligible articles were identified that reported both β-carotene and retinol levels in the two biological fluids at this lactation stage.

Colostrum was reportedly enriched in vitamin A content compared to serum [38,41,42,43,44,48]. A slight decrease in maternal circulating vitamin A levels is normally observed coinciding with an increase in colostrum vitamin A levels around late pregnancy-early gestation [99,100], indicating a higher retinol uptake by the mammary gland [101]. Since vitamin A is transferred to a limited extend over the placental barrier, this phenomenon takes place to provide the depleted newborn with the required amount of vitamin A to replenish stores and boost normal growth and healthy development [29].

Colostrum vitamin A values below 60 µg/dL were considered inadequate by four publications [41,42,43,44], and prevalence of colostrum vitamin A inadequacy was typically higher than the populations’ serum vitamin A inadequacy. The reference value set by the WHO in 1996 is ≤30 µg/dL breast milk and is based on dietary requirements of the infant to provide enough to prevent subclinical deficiency in the first six months of life [62]. This concentration, however, allows for little or no liver storage of vitamin A. As the infant is born with depleted vitamin A stores and consumes a lower milk volume in the first five days [102], and vitamin A levels in colostrum are typically higher compared to mature milk [63,103], this lactation period requires a higher reference value indicative of vitamin A-sufficient colostrum. Though, the employed value of 60 µg/dL is arbitrary as no explanation of criteria is given in the respective publications [41,42,43,44], nor in the referred sources [61,63].

### 4.6. Vitamin E

Conflicting results are reported on the differences between serum and colostrum mean α-tocopherol concentrations, with higher colostrum levels found in three studies [36,38,39], comparable levels in one [42], and slightly lower levels in two studies [41,40], as compared to serum. Differing general and obstetric characteristics between the studied populations may underlie these discrepancies [104].

However, the difference in mean values was insignificant for most studies, with high variations in colostrum α-tocopherol content [36,38,40,41,42]. Only Clemente et al. found significantly higher colostrum α-tocopherol in two of three groups. This is in line with the observed decrease in serum α-tocopherol and considerate increase in colostrum α-tocopherol at late gestation and early lactation [100,105,106].

All eligible articles reported colostrum α-tocopherol content as more variable than serum levels (Figure 2), possibly due to colostrum’s sensitivity to variation in dietary α-tocopherol ingestion and a tight homeostatic control of α-tocopherol in the blood. The absence of correlation between colostrum and serum vitamin E [39,40,41,42] points to selective transport mechanisms of this vitamin to the milk, independent of blood concentrations. The mechanism of α-tocopherol transfer from blood into milk in humans is unknown. α-Tocopherol is stored in the liver bound to α-tocopherol transfer proteins (α-TTPs). The extrahepatic excretion and subsequent incorporation of this complex in the outer layer of lipoproteins allows transport and distribution throughout the body. Evidence in other mammals indicates that tissue α-tocopherol uptake occurs via several pathways, mainly by LPL and lipoprotein receptors. In the mammary gland, LPL activity regulates the uptake of α-tocopherol during late pregnancy and lactation, as tissue concentration of α-tocopherol parallels LPL activity in the mammary gland [94,107]. Alternatively, α-tocopherol could reach the mammary tissue by means of lipoprotein receptors for low-density lipoproteins (LDL), and/or be transported by scavenger receptor class B, type 1 (SCARB1, formerly known as SR-B1, involved in the supply of α-tocopherol by means of the high-density lipoprotein (HDL) receptors, potentially involving intracellular membrane receptors for α-TTP in the mammary epithelium [108,109]. An α-TTP-dependent mechanism in the mammary gland cannot be excluded, as mammary gland α-TTP can facilitate α-tocopherol secretion into milk. Jensen et al. showed that the daily α-tocopherol milk secretion can be described using Michaelis-Menten kinetics, which applies for carrier-mediated transport across membranes [99]. Passive transfer along with triacylglycerols appears to be excluded as the lipid profile of milk completely differs from that of vitamin E, and the daily quantity excreted is independent of amount of milk and milk fat yield [101,110]. This supports the hypothesis of protein-mediated α-tocopherol transfer from blood to milk [99].

### 4.7. Serum Retinol Affects Colostrum α-Tocopherol

Biochemical analysis of retinol-sufficient (≥30 µg/dL) lactating women showed serum retinol concentrations to inversely correlated with α-tocopherol colostrum levels (*r* = −0.28, *p* = 0.008), but this was not observed for mothers with inadequate retinol serum levels below 30 µg/dL by de Lira et al. [42]. This indicates the aspect of competition for retinol and α-tocopherol transfer over the blood-milk barrier. The modulating effect of retinol on α-tocopherol was only observed under conditions of high vitamin A intake [29]. The mechanism behind this phenomenon has not been fully explained. Evidence indicates that retinoic acid isomers may have a negative influence on the production of α-TTPs, which reduces the incorporation of α-tocopherol in LDL, thereby affecting its plasma concentrations and bodily distribution [111]. In addition, it is suggested that retinoids reduce hepatic mRNA expression of apoliprotein A-1 that is present in HDL, which transport α-tocopherol to tissues [112]. Moreover, SCARB1 also has a role in role in retinol transport [113,114]. The negative influence of serum retinol on colostrum α-tocopherol concentrations that were observed in this study may thus be explained by mechanisms in the mammary gland, involving α-TTP [29,105], as well as SCARB1. The negative impact of vitamin A supplementation on α-tocopherol colostrum levels is confirmed by the observation that 24 h after a single supplementation with 20,000 IU liquid retinyl palmitate, in a study population with a vitamin A deficiency rate of 5.7% (<20 µg/dL), colostrum α-tocopherol levels significantly decreased by 16.4% [38]. Regrettably, in this study, no post-supplemental blood samples were obtained to consolidate the likely increase in systemic retinol levels, hence they did not directly report an association between serum and colostrum. A decrease in colostrum α-tocopherol upon vitamin A (retinyl-palmitate) supplementation is not always observed, but invariably this concerns co-supplementation with α-tocopherol. That maternal supplementation with a mega-dose of retinyl palmitate raises vitamin A, but decreases vitamin E bioavailability in colostrum, exemplifies interactions between different vitamins present in serum and colostrum, and emphasises the importance of assessing the relationship between different vitamins in these two biological fluids. 

## 5. Conclusions

Our review identifies a significant gap in the available information on the quantitative relationship between serum/plasma vitamin content and colostrum vitamin composition in healthy mothers. Based on our predefined criteria, the restricted selection of vitamins A, C, D, E, and K have been studied, with an emphasis on vitamin A and E in the Brazilian population. Within this scope, interactions between different vitamins have been studied to a very limited extent in studies to date, given solely the effect of serum vitamin A on colostrum vitamin E levels was reported in one paper. Colostrum appears to be enriched in vitamins A, C, and K compared to maternal serum, possibly indicative of active mammary gland uptake. Interestingly, correlation between the two biological fluids for vitamins D, K, E, and A is lacking, possibly indicating selective vitamin transport mechanisms of the mammary gland that are independent of plasma vitamin concentration. Inter-individual and inter-study variability in colostrum’s vitamin content points towards its sensitivity to external factors. Research is required to validate single-article findings on vitamin C, D, and K levels, and to further explore this relationship for other lipid- and water-soluble vitamins. Future studies on the relationship between maternal vitamin status and colostrum vitamin composition should be quantitative in nature, and should adopt standard methods for milk sampling, storage and analysis, and cover on healthy, normal, populations of different ethnic backgrounds.

## 6. Limitations and Strengths of the Performed Research

The review procedure as described by the PRISMA statement of Systematic Reviews was only followed up to the full-text screening stage. During the full-text screening process, we discovered the review scope to be too vast. Our measures taken to render this review feasible within the time frame of the project do not strictly correspond to a systematic approach, and are therefore considered as a limitation of the present review. In addition, no quality assessments of the included publications was performed along the lines of quality assessment forms for cross-sectional studies and randomised clinical trials, though the limitations regarding study populations and reported results were reviewed. Moreover, due to our pre-defined and well informed inclusion and exclusion criteria, the vast majority of vitamin B papers were excluded from the review, such as the exclusivity of breastfeeding and the socioeconomic status of the enrolled subjects. Furthermore, in some of the vitamin B studies, maternal plasma/serum/milk micronutrient status were not measured simultaneously and/or determined in the same individual and some studies focused on the analogs of vitamin B rather than the actual vitamin B itself. However, to our knowledge, no review exists on maternal vitamin state and colostrum vitamins in average-to-high socio-economic and healthy populations, for which this review represents a novel compilation of valuable data regarding the immediate postpartum period.

### Recommendations

Circulating levels of vitamins can be determined in large population samples with relative ease, and are a relevant source for vitamins in colostrum, hence it is relevant to establish the relation between circulating and colostrum vitamin levels. While serum/plasma levels for vitamin C and D are an appropriate biomarker [115,116], this is less convincing for the other vitamins. For vitamin K, no convincing biomarkers are known, with circulating levels of vitamin K (phylloquinone) being a biomarker of especially short-term intake in adults [117]. Similarly for vitamin E, serum vitamin E is considered to be a non-sensitive biomarker for vitamin E status [118]. For vitamin A, serum levels are under tight homeostatic control and hepatic vitamin A levels are considered the marker of vitamin A status, because in healthy individuals, 70–90% of the retinol in the body is stored in the liver. For vitamin A, and possibly for other vitamins and mineral not discussed in this review, it is therefore recommended to assess relevant status biomarkers in addition to serum levels. Furthermore, it would be interesting and relevant to compare the status in healthy non-pregnant non-lactating women and healthy lactating women. Finally, in view of development of targeted nutritional interventions, non-radioactive tracer studies may be used to reveal to which extend acute intake of vitamins ends up in colostrum.

In order to complement the current evaluation on vitamins, the search term can be modified to target the relationship of maternal essential mineral status in blood and colostrum through a systematic review. Herewith, one can generate a complete overview of micronutrient composition of these two biological fluids in separate publications. Furthermore, it would be interesting to evaluate available information on this relationship at later stages of lactation. The revealed knowledge gap suggests to complement hypothesis-driven, reductionist approaches (studying one or only a few vitamins in isolation) with more explorative, systemic approaches to measure the complete vitamin profile of maternal serum and colostrum. In order to address this knowledge gap, it is recommended to generate a comprehensive overview of the entire vitamin profile in maternal blood and colostrum from healthy, non-deficient individuals. Ideally, a cohort study should be performed utilising the highly specific and sensitive quantification method of liquid chromatography–tandem mass spectrometry. Ideally, internal standards for each vitamin should be taken to verify vitamin concentrations in both matrices. These methods have recently become available in the context of vitamin profiling and metabolomics [119,120,121]. Herewith, one can possibly elucidate interactions between vitamins regarding transport from the blood to the milk, contributing to physiological understanding of the multifaceted exocrine functioning of the mammary gland, and giving rise to novel ideas and hypotheses.

In addition to maternal status and colostrum vitamin composition, it would be interesting to simultaneously track the vitamin status and health outcomes of the respective healthy newborns. Bioavailability of vitamins in the maternal milk matrix might be higher as compared to supplements or dietary intake, especially for lipid-soluble vitamins. The latter are packed in and/or accompanied by lipid globules present in milk, and fat-rich food is known to enhance intestinal absorption of lipid-soluble vitamins. The current AIs for several vitamins for the first six months of life are calculated based on the average intake and nutritional composition of later human milk [19,62]. Vitamin A and E adequacy levels in colostrum were arbitrarily set by authors of eligible publications due to a lack of scientific evidence addressing this postpartum period. Since the intake volume of breast milk varies across lactation [102] and colostrum greatly differs in nutritional (fat) composition from later milk [122], different recommendations and colostrum vitamin-deficiency cut-off values possibly apply to the immediate post-partum phase to meet infant requirements.

## Figures and Tables

**Table 1:** Dietary Reference Intakes (DRIs): Recommended Dietary Allowances (RDA) and Adequate Intakes (AI) of vitamins during lactation and the first six months of life. Values as established by the Food and Nutrition Board, Institute of Medicine, National Academies, USA.

Life Stage Group	Vit A (µg/day) ^a^	Vit D (µg/day) ^b,c^	Vit E (mg/day) ^d^	Vit K (µg/day)	Vit C (mg/day)	Thiamin (mg/day)	Riboflavin (mg/day)	Niacin (mg/day) ^e^	Vit B6 (mg/day)	Folate (µg/day) ^f^	Vit B12 (µg/day)	Pantothenic Acid (mg/day)	Biotin (µg/day)	Choline (mg/day) ^g^
**Infants**														
**0–6 month**	400 *	5 *	4 *	2.0 *	40 *	0.2 *	0.3 *	2 *	0.1 *	65 *	0.4 *	1.7 *	5 *	125 *
**Lactating women**														
**14–18 year**	1200	15	19	75 *	115	1.4	1.6	17	2.0	500	2.8	7 *	35 *	550 *
**19–30 year**	1300	15	19	90 *	120	1.4	1.6	17	2.0	500	2.8	7 *	35 *	550 *
**31–70 year**	1300	15	19	90 *	120	1.4	1.6	17	2.0	500	2.8	7 *	35 *	550 *

SOURCES: [19,32,33,34]; the table is based on DRI tables published by the Institute of Medicine; NOTE: The RDA values reflect the intake that meets the nutrient need of almost all (97–98%) individuals in a group. For healthy infants receiving human milk, the Adequate Intake (AI) of a vitamin represents the mean intake. For other life stages, the AI is believed to cover the needs of all healthy individuals in the group, but lack of data or uncertainty in the data prevent the ability to specify the percentage of individuals covered by this intake with confidence; * Adequate Intake (AI); ^a^ As retinol activity equivalents (RAEs). 1 RAE = 1 μg retinol, 12 μg β-carotene, 24 μg α-carotene, or 24 μg β-cryptoxanthin. The RAE for dietary provitamin A carotenoids is two-fold greater than retinol equivalents (RE), whereas the RAE for preformed vitamin A is the same as RE; ^b^ As cholecalciferol. 1 µg cholecalciferol = 40 IU vitamin D; ^c^ In the absence of adequate exposure to sunlight; ^d^ As α-tocopherol. α-Tocopherol includes RRRα-tocopherol, the only form of αtocopherol that occurs naturally in foods, and the 2R-stereoisomeric forms of α-tocopherol (RRR-, RSR-, RRS-, and RSS-α-tocopherol) that occur in fortified foods and supplements; ^e^ As niacin equivalents (NE). 1 mg of niacin = 60 mg of tryptophan; 0–6 months = preformed niacin (not NE); ^f^ As dietary folate equivalents (DFE) 1 DFE = 1 µg food folate = 0.6 µg of folic acid from fortified food or as a supplement consumed with food = 0.6 µg of a supplement taken on an empty stomach.; ^g^ Although AIs have been set for choline, there are few data to assess whether a dietary supply of choline is needed at all stages of the life cycle, and it may be that the choline requirement can be met by endogenous synthesis at some of these stages.

**Table 2:** Predefined and restricted screening criteria. Along the lines of these inclusion and exclusion measures, titles, abstracts, and full texts were screened for eligibility.

Inclusion	Exclusion
■ Validation studies in (clinically) healthy (as defined by investigators) human subjects ■ Studies conducted in underdeveloped, developing and developed countries. ■ Population with average and high socioeconomic statuses ■ Studies involving non-pregnant lactating and exclusively breastfeeding mothers ■ Studies where maternal micronutrient status was recorded while lactating ■ Studies that have measured maternal plasma and milk micronutrient status simultaneously within the same individuals ■ Studies that have measured maternal plasma and milk micronutrient status simultaneously ■ Lactating period of 0–12 months ■ Dietary intervention studies focussed on micronutrient supplementation ■ Primary articles	■ Animal and in vitro studies ■ Studies that have focused on diseased, unhealthy, under- or malnourished (micronutrient deficient) subjects ■ Chronic metabolic or immunological disorders/HIV ■ Mothers with unhealthy infants, that were preterm (<37 weeks gestation) or had low birth weight (<2500 g) ■ Studies with no information on health status of study population ■ Studies conducted in low socioeconomic populations ■ Population that is smoking, uses oral contraceptives, consumes alcohol, or with a BMI > 25 pre-pregnancy ■ Any duplicate publications ■ Articles lacking full texts ■ Conference proceedings without published full text articles ■ Studies assessing only one of the two biological fluids ■ Partial breastfeeding (weaning) ■ Studies focusing on drugs/toxic metals (inorganic minerals arsenic, mercury, lead), contaminants, pollutants in human milk ■ Review or secondary articles

**Table 3:** Characteristics of publications on the relationship between maternal vitamin state and colostrum vitamin composition.

First Author, Year [Ref]	Country	Study Design and Participants	Colostrum and Blood Samples	Method	Main Results	
					Maternal Serum (Mean ± SD)	Colostrum (Mean ± SD)
Melo, 2017 [36]	Brazil	RCT; 99 mothers; mean ± SD (range) age: 24 ± 6 (18–40) year; divided in CG (*n* = 39) and IG (*n* = 60); no maternal and infant pathologies, full-term healthy delivery. Mixed population based on socio-economic (marriage, education, income) and obstetric characteristics (maternal BMI, parity, way of delivery). Limitations: 51% normal pre-pregnancy weight; partly low SES; effect of these parameters on baseline vitamin E not assessed, postpartum day unknown.	Colostrum (2 mL) collected through manual expression of one breast, at end of feeding; foremilk was discarded; blood (5 mL) collected by venipuncture; in morning after overnight fast; baseline measurement; vitamin E was measured.	HPLC-UV spectro-photometry	α-Tocopherol:CG: 1066.6 ± 287.7 mg/dL *; IG: 1159.6 ± 350 mg/dL *; not significantly different between groups (*p* = 0.41); overall in 3.0% (*n* = 3) α-tocopherol deficiency (<499.6 mg/dL) [37]	α-Tocopherol: CG: 1509.3 ± 793.7 mg/dL *; individual analysis: 33% (*n* = 13) colostrum α-tocopherol levels too low to meet infant daily requirements (4 mg/day). IG: 1452.9 ± 808.6 mg/dL *; Individual analysis: 30% (*n* = 18) colostrum α-tocopherol levels too low to meet infant daily requirements (4 mg/day). α-Tocopherol not significantly different between groups (*p* = 0.74).
Grilo, 2016 [38]	Brazil	RCT; 88 mothers; mean ± SD age: 24 ± 7 year; divided in IG (*n* = 44) and CG (*n* = 44); total sample shows diversity in maternal, obstetric and newborn characteristics; similar for the two groups (*p* > 0.05). Limitations: 40% had pre-gestational BMI > 25; 40% had insufficient gestational weight gain; 14% low birth weight; effect of these parameters on baseline vitamin E not assessed.	Colostrum (2 mL) and blood (5 mL) collected after overnight (8–12 h) fast, 1 d postpartum; stored −20 °C (0–4 d) until analyses; baseline measurement; vitamin A and E were measured.	HPLC-UV spectro-photometry	Retinol: CG 44.8 ± 16.4 µg/dL; IG 48.3 ± 15.4 µg/dL; not significantly different between groups (*p* > 0.05). Total population 46.4 ± 15.9 µg/dL; 5.7% deficient (<20 µg/dL).	Retinol: CG 101.3 ± 63.3 µg/dL; IG: 102.1 ± 47.7 µg/dL; not significantly different between groups (*p* > 0.05)
					α-Tocopherol: CG 1023.6 ± 380.4 µg/dL; IG: 1059.0 400.3 µg/dL; not significantly different between groups (*p* > 0.05); Total population 1023.6 ± 380.4 µg/dL; 9.1% deficient (<516 µg/dL)	α-Tocopherol: CG 1189.4 ± 660.6 µg/dL; IG: 1259.2 ± 608.2 µg/dL; not significantly different between groups (*p* > 0.05).
Clemente, 2015 [39]	Brazil	RCT; 109 mothers; mean ± SD age: 24.1 ± 5.6 year; control group (CG; *n* = 36), natural supplemented group (G_NAT_; 400 IU of *RRR*-α-tocopherol; *n* = 40) and synthetic supplemented group (G_SIN_; 400 IU of all-rac-α-tocopherol; *n* = 33).	Colostrum (2 mL) collected through manual expression; foremilk was discarded; blood (5 mL) collected by venipuncture; after an overnight fast, ~12 h postpartum; baseline measurement; vitamin E was measured.	HPLC-UV spectro-photometry	α-Tocopherol: CG 1016 ± 52 µg/dL; G_NAT_ 1236 ± 51 µg/dL; G_SIN_ 1083 ± 61 µg/dL; not significantly different between groups (*p* = 0.546).	α-Tocopherol: CG 1665.2 ± 160.2 µg/dL; G_NAT_ 1387.1 ± 176.5 µg/dL; G_SIN_ 1802 ± 208.1 µg/dL; no difference between groups in the mean (*p* = 0.253) or the variance (*p* = 0.767).
Thijssen, 2017 [40]	The Netherlands	RCT; 31 mothers; no drug use or abuse, maternal/infant gastrointestinal dysfunction or low (<50 kg) or high (>90 kg) maternal body weight; randomly assigned to four treatment arms; no difference in baseline values between groups. Limitations: limited information on population characteristics.	Colostrum (5–10 mL) collected through manual pump device from the breast not used for the previous feed, first 10 mL was discarded; blood collected; between 8–11 am, 4 d postpartum; stored at −70 °C until analysis; baseline measurement; vitamin K and E were measured.	Vitamin K: fluorescence detection, HPLC separation and post-column reduction (Zn-column) Vitamin E: HPLC-UV spectro-photometry	Phylloquinone (plasma): Ranged from 0.5 to 7.2 nmol/L 95% CI (1.89, 3.28); (mean ± SD): 2.62 ± 1.91 nmol/L (1.18 ± 0.86 µg/L) (*n* = 31)	Phylloquinone: 5.84 ± 2.31 nmol/L (2.63 ± 1.04 µg/L) (*n* = 31)
					Colostrum:plasma concentration ratio phylloquinone: 3.97 ± 3.11 (*n* = 31)	
					Menaquinone-4 (plasma): 0.25 ± 0.16 nmol/L (0.11 ± 0.07 µg/L); 95% confidence interval (0.14, 0.37); detected in 32.3% (*n* = 10) of the samples;	Menaquinone-4: 3.18 ± 1.53 nmol/L (1.41 ± 0.68 µg/L) (*n* = 31)
					Colostrum:plasma concentration ratio Menaquinone-4: 14.83 ± 8.3 (*n* = 10)	
					Plasma α- and γ- tocopherol: 30.4 ± 6.1 µmol/L (*n* = 31)	α- and γ- tocopherol: 26.5 ± 18.5 µmol/L (*n* = 31)
					Colostrum:plasma concentration ratio α- and γ- tocopherol: 0.97 ± 0.62 (*n* = 31)	
Garcia, 2010 [41]	Brazil	Cross-sectional study; 73 mothers, free of pathologies, sufficient birthweight; divided in intervention group (IG; *n* = 36) and control group (CG; *n* = 37); age 14–18 year (*n* = 17); 19–36 year (*n* = 56). Limitations: limited information on population characteristics.	Colostrum (2 mL) collected by manual pressure of single breast not previously suckled; first ejection discarded; stored at −20 °C until analyses; 5 mL blood collected after overnight fast up to 16 h postpartum; baseline measurement; vitamin A and E were measured.	HPLC-UV spectro-photometry	Retinol (plasma): 1.77 ± 0.50 µmol/L (50.72 ± 14.33 µg/dL) (*n* = 73)	Retinol: CG: 2.6 ± 1.9 µmol/L (74.49 ± 54.44 µg/dL); IG 2.4 ± 2.4 µmol/L (68.77 ± 68.77 µg/dL); not significantly different between groups (*p* > 0.05)
					No correlation between colostrum and plasma retinol (*r* = 0.39 and *p* = 0.74)	
					α-Tocopherol (plasma): 30.81 ± 6.46 µmol/L (1326.9 ± 278.2 µg/dL) (*n* = 73)	α-Tocopherol: 28.27 ± 22.29 µmol/L (1217.4 ± 959.9 µg/dL) (*n* = 73)
					No correlation between colostrum and plasma α-tocopherol (*r* = 0.74 and *p* = 0.54)	
De Lira, 2013 [42]	Brazil	Cross-sectional study; 103 mothers, age range 14–41 year (24 ± 7 year), free of associated pathologies; mixed population based on obstetric, maternal and newborn parameters, but none of the variables was associated with retinol and α-tocopherol levels (*p* > 0.05). Subgroup (*n* = 87) without serum retinol deficiency (≥30 µg/dL). Subgroup (*n* = 88) without serum α-tocopherol deficiency [37]. Limitations: 36% pre-gestational BMI > 25, 20% exceeded gestational weight gain; 24% preterm deliveries; 40% and 7% infants had insufficient and excessive birth weight, respectively.	Colostrum (2 mL) collected after overnight fast for three consecutive days (1–3 d postpartum) to establish colostrum pool; blood (5 mL) collected after overnight fast 1 d postpartum; vitamin A and E were measured.	HPLC-UV spectro-photometry	Retinol: 1.49 ± 0.4 µmol/L (42.69 ± 11.46 µg/dL) (*n* = 103); 15.5% inadequate. Subgroup retinol: 1.61 ± 0.35 µmol/L (46.13 ± 10.03 µg/dL) (*n* = 87)	Retinol: 2.18 ± 0.8 µmol/L (62.46 ± 22.92 µg/dL) (*n* = 103) Subgroup retinol: 2.25 ± 0.79 µmol/L (64.47 ± 22.64 µg/dL) (*n* = 87); colostrum retinol deficiency (<60 µg/dL) in 34% (*n* = 30)
					No correlation between serum and colostrum retinol (*p* = 0.11, *r* = 0.15) (*n* = 103)	
					α-Tocopherol: 26.4 ± 8.0 µmol/L (1137.0 ± 344.5 µg/dL) (*n* = 103); 16% inadequate (<500 µg/dL). Subgroup α-tocopherol : 28.7 ± 8.08 µmol/L (1236.0 ± 348.0 µg/dL) (*n* = 88)	α-Tocopherol: 26.1 ± 12.8 µmol/L (1124.0 ± 551.2 µg/dL) (*n* = 103); Subgroup α-tocopherol: 28.24 ± 16.1 µmol/L (1216.2 ± 693.4 µg/dL) (*n* = 88); colostrum α-tocopherol deficiency in 44% (<26.1 µmol/L).
					No correlation between serum and colostrum α-tocopherol (*r* = −0.12, *p* = 0.22) (*n* = 103). Inverse correlation between serum retinol and colostrum α-tocopherol (*r* = −0.28, *p* = 0.008) in retinol adequate subgroup (*n* = 87); No correlation between serum retinol and colostrum α-tocopherol in inadequate fraction of the population (*n* = 16).	
Gurgel, 2017 [43]	Brazil	Cross-sectional study; 100 mothers; mean age 28.6 year; free of morbidities and healthy deliveries; CG no supplement (*n* = 25); intervention groups (*n* = 25 per group) had taken daily supplements from 16 wk gestation until delivery; IG_1_ (1500 IU retinol +1500 IU β-carotene, 112.5% RDI); IG_2_ (2700 IU β-carotene, 101% RDI); IG_3_ (2664 IU retinol, 99.9% RDI); maternal age, years of education, occupation and income was not significantly different between groups (*p* > 0.05); average vitamin A dietary intake in last trimester of pregnancy was not significantly different between groups (*p* > 0.05). Limitations: effect of these parameters on vitamin A levels not assessed, postpartum day unknown.	Colostrum (500 µL) collected through manual expression of a single breast at the start and end of the breastfeeding; blood (5 mL) collected through venipuncture; under fasting conditions; stored −20 °C until analyses; vitamin A was measured.	HPLC-UV spectro-photometry	Retinol: CG: 45.4 ± 11.8 µg/dL (1.58 ± 0.41 µmol/L), serum retinol inadequacy (<20 µg/dL) in 12% (*n* = 3); IG_1_: 46.5 ± 13.3 µg/dL (1.62 ± 0.46 µmol/L); IG_2_: 43.5 ± 13.7 µg/dL (1.52 ± 0.48 µmol/L); IG_3_: 47.5 ± 13.0 µg/dL (1.65 ± 0.45 µmol/L); no vitamin A inadequacy in supplemented groups; Serum retinol levels not significantly different between groups (*p* > 0.05).	Retinol: CG 96.6 ± 53.5 µg/dL (3.37 ± 1.87 µmol/L), colostrum retinol inadequacy (<60 µg/dL) in 20% (*n* = 5); IG_1_: 126.1 ± 48 µg/dL (4.40 ± 1.68 µmol/L), inadequacy in 4% (*n* = 1); IG_2_: 89 ± 61.9 µg/dL (3.11 ± 2.16 µmol/L), inadequacy in 40% (*n* = 10); IG_3_: 136.8 ± 51.7 µg/dL (4.47 ± 1.80 µmol/L), inadequacy in 4% (*n* = 1); Colostrum retinol levels significantly different between IG_2_ and IG_3_ (*p* < 0.05).
Da Silva Ribeiro, 2010 [44]	Brazil	Cross-sectional study; 86 mothers; mean ± SD (range) age: 25.4 ± 5.8 (18–40) year; free of pathologies with full term pregnancy; data were analysed for the entire group, as well as for groups divided according to the predominant source of dietary vitamin A (group A >50% preformed vitamin A (*n* = 37) and group B (>50% pro-vitamin A carotenoids (*n* = 49)); cut-off value retinol inadequacy colostrum ≤ 60 µg/dL; no differences in general characteristics (age, newborn birth weight, gestational age) between groups. Limitations: No information on maternal BMI.	Colostrum (1–3 mL) collected by manual expression from single full breast not suckled in previous feed; first milk ejection discarded; 5 mL blood sample by venipuncture; after overnight fast, up to 16 h postpartum; stored −20 °C until analyses; vitamin A was measured.	HPLC-UV spectro-photometry	Retinol: Group A: 1.4 ± 0.4 µmol/L, inadequacy in 5.4% (≤0.70 µmol/L or ≤ 20 µg/dL); Group B: 1.2 ± 0.6 µmol/L, inadequacy in 12.2%. A sig. higher than B (*p* = 0.033). Total population (*n* = 86): 1.3 ± 0.51 µmol/L (28 ± 14.6 µg/dL), below adequacy in 9.3%.	Retinol: Group A: 3.4 ± 1.7 µmol/L; inadequacy (<2.1 µmol/L) in 24.3%; Group B: 3.6 ± 1.9 µmol/L, inadequacy in 20.4%; inadequacy similar in both groups. Total population (*n* = 86): 3.5 ± 1.9 µmol/L (100.3 ± 54.4 µg/dL) below adequacy in 22.1%.
Cancela, 1986 [45]	France	Cross-sectional study; 12 mothers, full term pregnancy; data of *n* = 1 was individually analysed because of vitamin D supplementation (7–14 d postpartum). Limitations: lack of information on population characteristics; pooled colostrum samples (1–3 d postpartum).	Colostrum (>5 mL) collected by manual pump expression at the end of first morning feed; blood samples obtained; 3–5 d postpartum, stored −20 °C until analyses, vitamin D (25OHD and vitamin D in colostrum and 25OHD and 1,25-(OH)_2_D_3_ in serum) was measured.	HPLC-UV spectro-photometry, competitive protein binding assay [46]	25OHD (mean ± SEM): 22.00 ± 2.61 µmol/L (*n* = 11); 20.0 µmol/L (*n* = 1) 1,25-(OH)_2_D_3_ (mean ± SEM): 0.194 ± 0.047 nmol/L (*n* = 11) 0.204 nmol/L (*n* = 1)	25OHD (mean ± SEM): 0.50 ± 0.11 nmol/L (*n* = 11); 0.44 nmol/L (*n* = 1) vitamin D (mean ± SEM): 0.89 ± 0.32 nmol/L (*n* = 11); 0.21 nmol/L (*n* = 1)
Ahmed, 2004 [47]	Bangladesh	Cross-sectional study; 26 mothers, age 18–32 year; mixed general characteristics (parity, income, BMI) did not influence milk vitamin C content; subgroup (*n* = 7) with eligible data. Limitations: influence of general characteristics were not assessed for vitamin C serum outcome of the subgroup; infant gestational weight was unspecified.	Colostrum (2 mL) collected by manual expression; blood (1 mL); 2 d postpartum; vitamin C was measured.	Centrifuga-tion; TCA and DTC; UV spectropho-tometry	Vitamin C: 0.44 ± 0.29 mg/dL (*n* = 7)	Vitamin C: 3.50 ± 0.49 mg/dL (*n* = 7)
Grilo, 2015 [48]	Brazil	Quasi-experimental study; 33 mothers, age 18–35 year, free of morbidities and no unhealthy, preterm births. Limitations: limited information on population characteristics, postpartum day unknown.	Colostrum (2 mL) collected by manual expression of a single breast at the beginning and end of breastfeeding; blood (5 mL) by venipuncture; after overnight fast; stored −20 °C until analyses, vitamin A was measured.	HPLC-UV spectropho-tometry	Retinol median (range): 37.3 (16.8 to 62.2) µg/dL	Retinol median (range): 46.8 (29.7 to 158.9) µg/dL
					No correlation between serum and colostrum retinol.	

RCT: Randomised Clinical Trial; IG:Intervention Group; CG: Control Group; CI: Confidence Interval, SD: Standard Deviation; RE: Retinol Equivalent; IU: International Unit; RDI: Recommended Daily Intake. * Units appear to be incorrect in the cited article.
